# Supplementary material for: Epigenetic priming improves salvage chemotherapy in diffuse large B-cell lymphoma via endogenous retrovirus-induced cGAS-STING activation
Source: Clin Epigenetics. 2023 May 3;15:75. doi: 10.1186/s13148-023-01493-x (PMC10155448; doi:10.1186/s13148-023-01493-x)
Supplement: Supplementary file 1 — Additional file 1: Figure S1. A-B. Schematics of the in vitro and in vivo treatment regimens, respectively. Figure S2. A-F. Six DLBCL cell lines were exposed to 5-azacytidine (0.3 µM) or PBS for three consecutive days followed by treatment with different doses of cisplatin for 48 hours. Cell viability measured on day 5 (normalized to that on day 3 for AZA- or PBS-treated cells) is shown. Figure S3. A, Gene expression profiles between the cisplatin-sensitive group and cisplatin-resistant group are shown using a heatmap. R: Resistant, S: Sensitive, RS: Re-sensitization. B, Gene expression profiles without or with 5-azacytidine treatment are shown using a heatmap. Figure S4. The expression level of ADAR1 determined using western blotting and RNAseq-based normalized expression data. Figure S5. Cell viability assay, OCI-LY1, SU-DHL2 and SU-DHL8 cells were treated with PBS, AZA, RTi, and AZA+RTi, followed by treatment with different doses of cisplatin for 48 hours. AZA: 5-azacytidine, RTi: reverse transcriptase inhibitor (delavirdine mesylate). Figure S6. The LINEs expression levels in each cell lines treated with PBS or 5-azacytidine were shown using a bar chat. Figure S7. A-B. OCI-LY1 and SU-DHL2 cells were treated with 0.3 µM 5-azacytidine for three consecutive days and 250 µM vitamin C once exposed for three days or only 0.3 µM 5-azacytidine or PBS for three consecutive days, followed by treatment with different doses of cisplatin for 48 hours. C-E, Subcutaneous tumor sizes, xenograft tumor images and tumor weights of xenografts from OCI-LY1 tumor-bearing models are shown. *p < 0.05, *p < 0.01 and ***p < 0.001 two-way ANOVA). F. The expression level of TET2 determined using RNAseq-based normalized expression data is shown in a heatmap. Figure S8. Schematic diagram showed the background of the current study. Epigenetic priming-induced chemosensitization is a potential solution for the unmet needs of salvage chemotherapy, however, its potential and mechanism to improve [file 13148_2023_1493_MOESM1_ESM.docx]

**
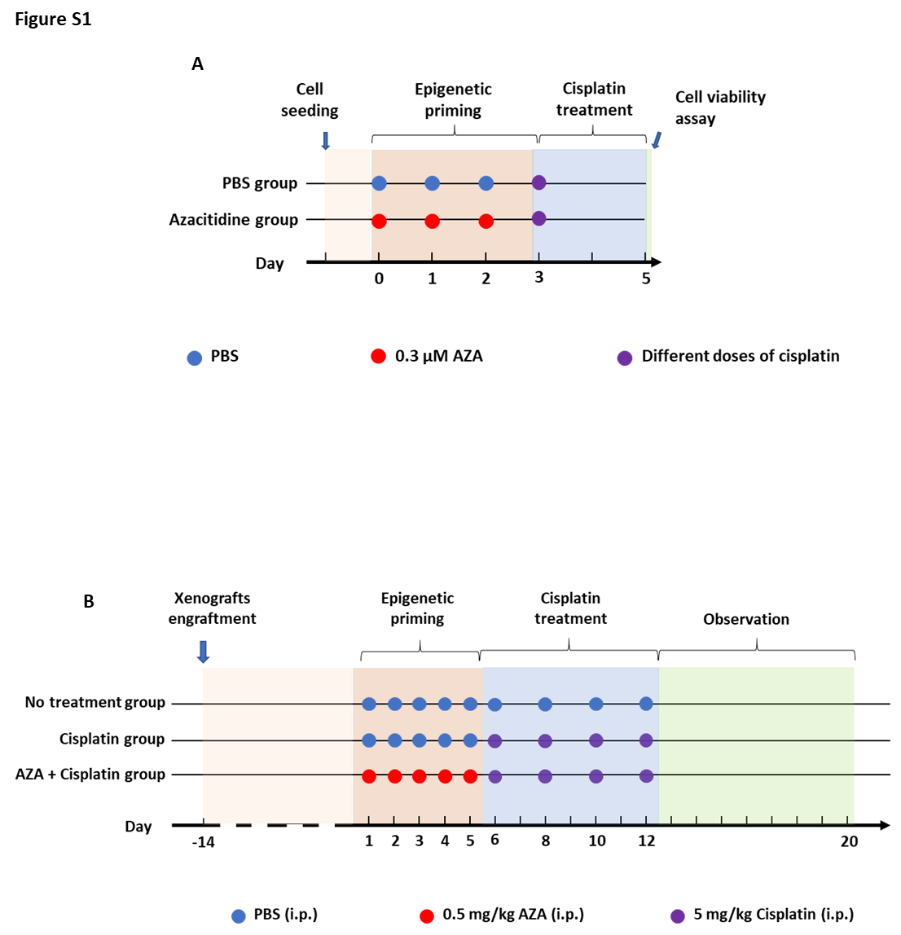
**

# Figure S1

A-B. Schematics of the in vitro and in vivo treatment regimens, respectively.


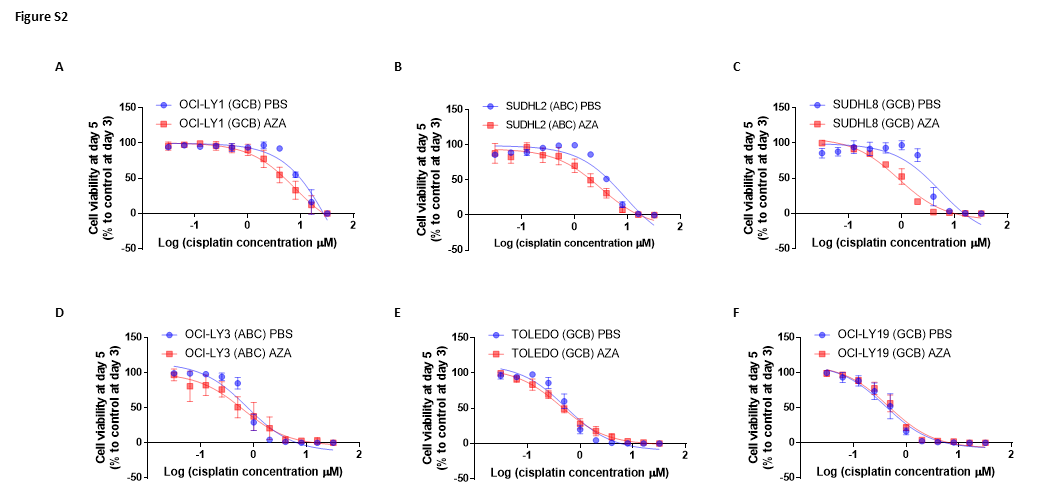


# Figure S2

# A-F. Six DLBCL cell lines were exposed to 5-azacytidine (0.3 µM) or PBS for three consecutive days followed by treatment with different doses of cisplatin for 48 hours. Cell viability measured on day 5 (normalized to that on day 3 for AZA- or PBS-treated cells) is shown.


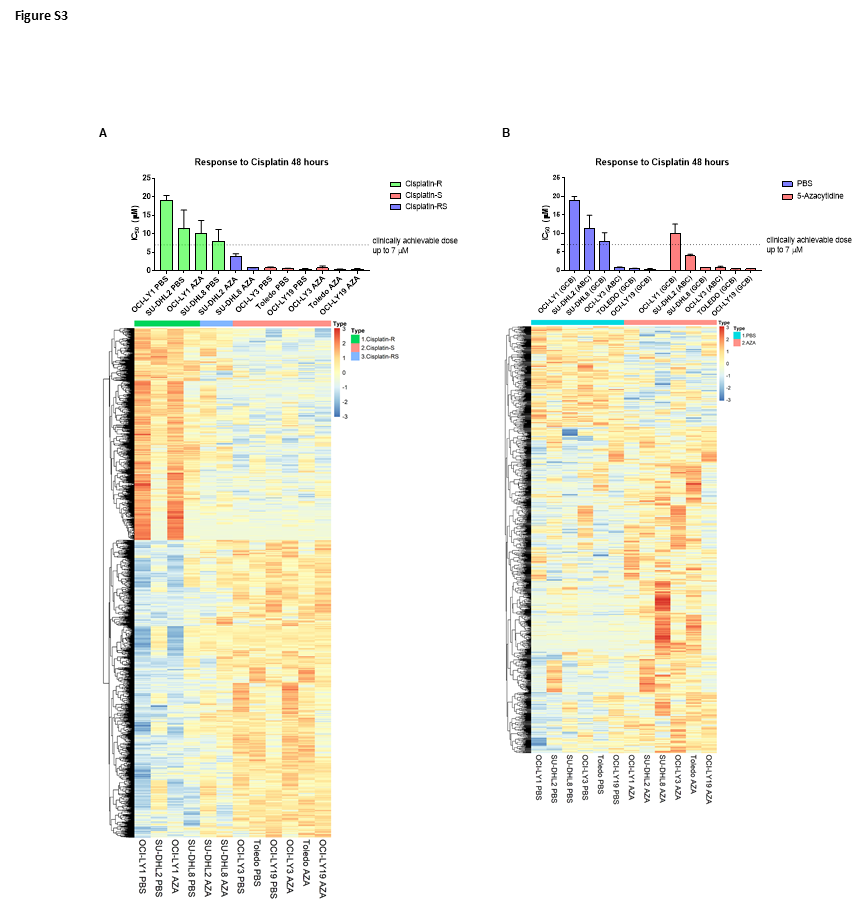


# Figure S3

A, Gene expression profiles between the cisplatin-sensitive group and cisplatin-resistant group are shown using a heatmap. R: Resistant, S: Sensitive, RS: Re-sensitization. B, Gene expression profiles without or with 5-azacytidine treatment are shown using a heatmap.


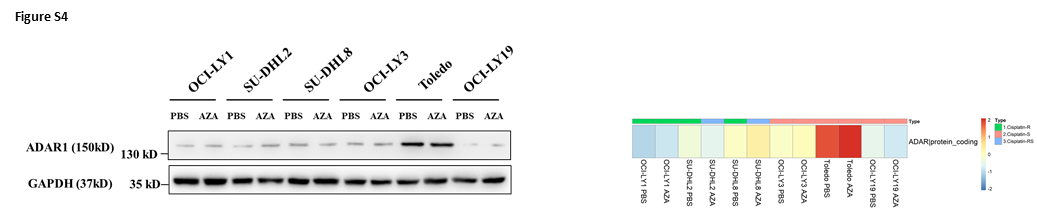


# Figure S4

# The expression level of ADAR1 determined using western blotting and RNAseq-based normalized expression data.

**
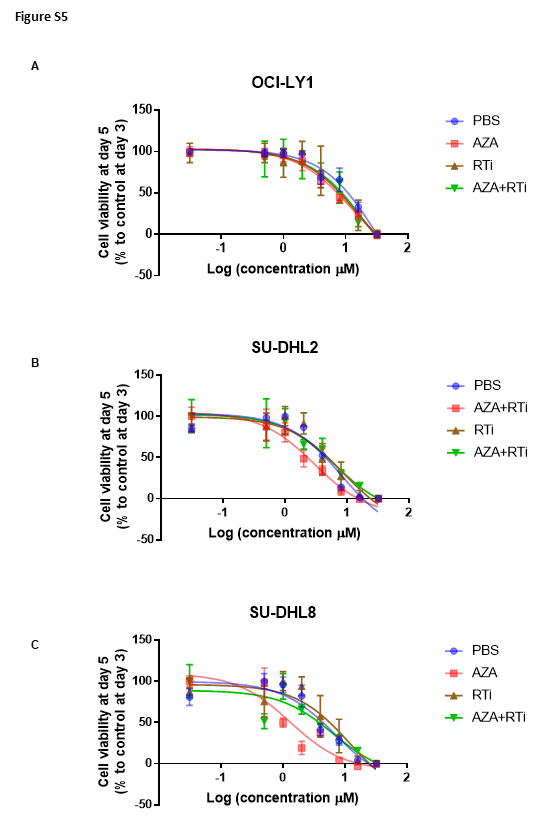
**

# Figure S5

# Cell viability assay, OCI-LY1, SU-DHL2 and SU-DHL8 cells were treated with PBS, AZA, RTi, and AZA+RTi, followed by treatment with different doses of cisplatin for 48 hours. AZA: 5-azacytidine, RTi: reverse transcriptase inhibitor (delavirdine mesylate).

#
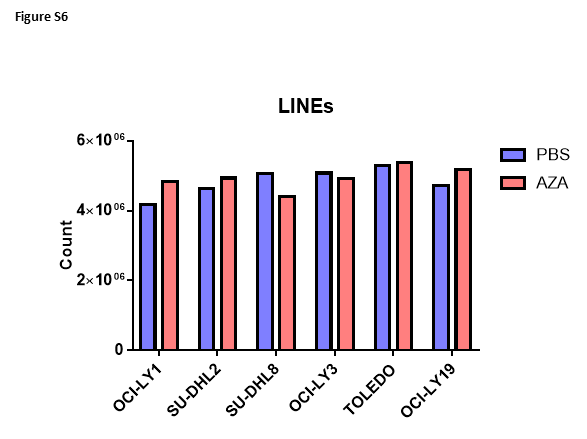


# Figure S6

# The LINEs expression levels in each cell lines treated with PBS or 5-azacytidine were shown using a bar chat.

**
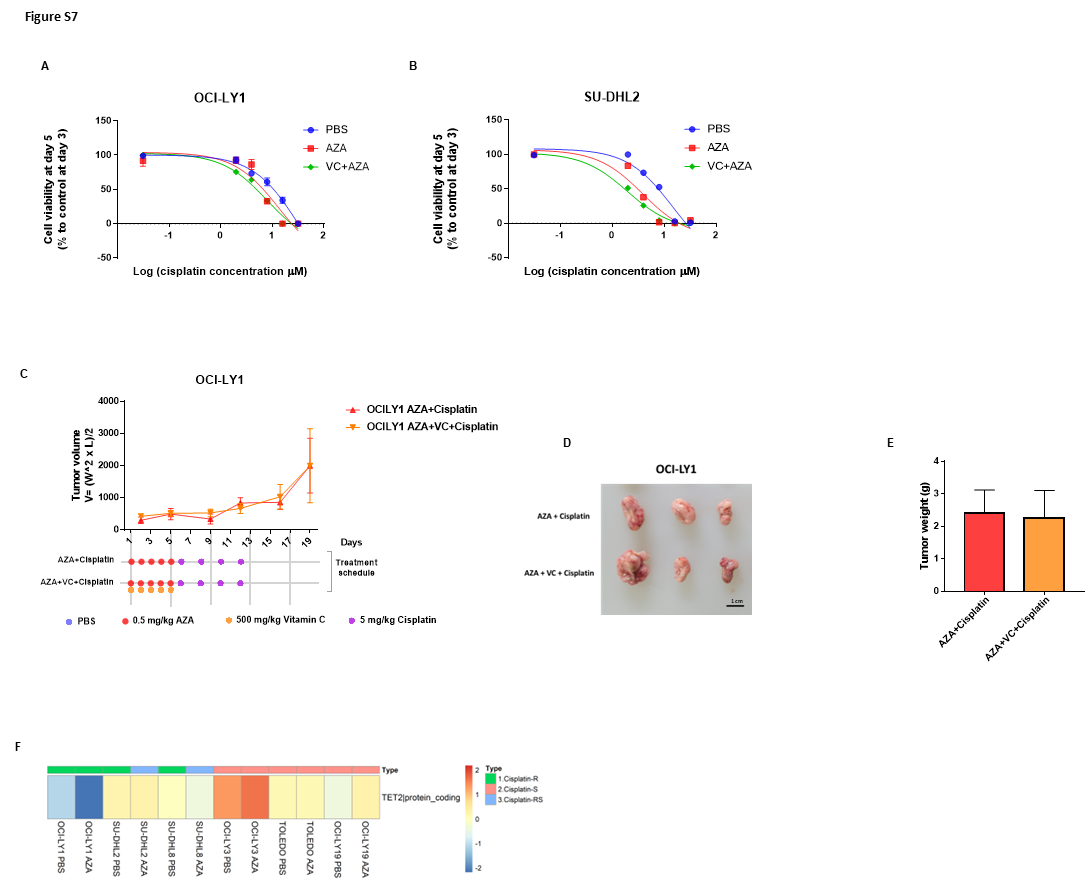
**

# Figure S7

# A-B. OCI-LY1 and SU-DHL2 cells were treated with 0.3 µM 5-azacytidine for three consecutive days and 250 µM vitamin C once exposed for three days or only 0.3 µM 5-azacytidine or PBS for three consecutive days, followed by treatment with different doses of cisplatin for 48 hours. C-E, Subcutaneous tumor sizes, xenograft tumor images and tumor weights of xenografts from OCI-LY1 tumor-bearing models are shown. **p* < 0.05, **p* < 0.01 and ****p* < 0.001 two-way ANOVA). F. The expression level of TET2 determined using RNAseq-based normalized expression data is shown in a heatmap.

#
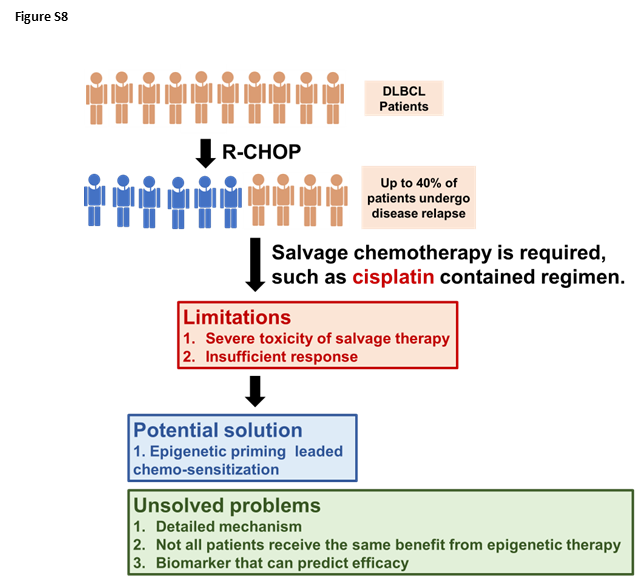


# Figure S8

# Schematic diagram showed the background of the current study. Epigenetic priming-induced chemosensitization is a potential solution for the unmet needs of salvage chemotherapy, however, its potential and mechanism to improve outcomes in relapsed/refractory DLBCL is unknow.


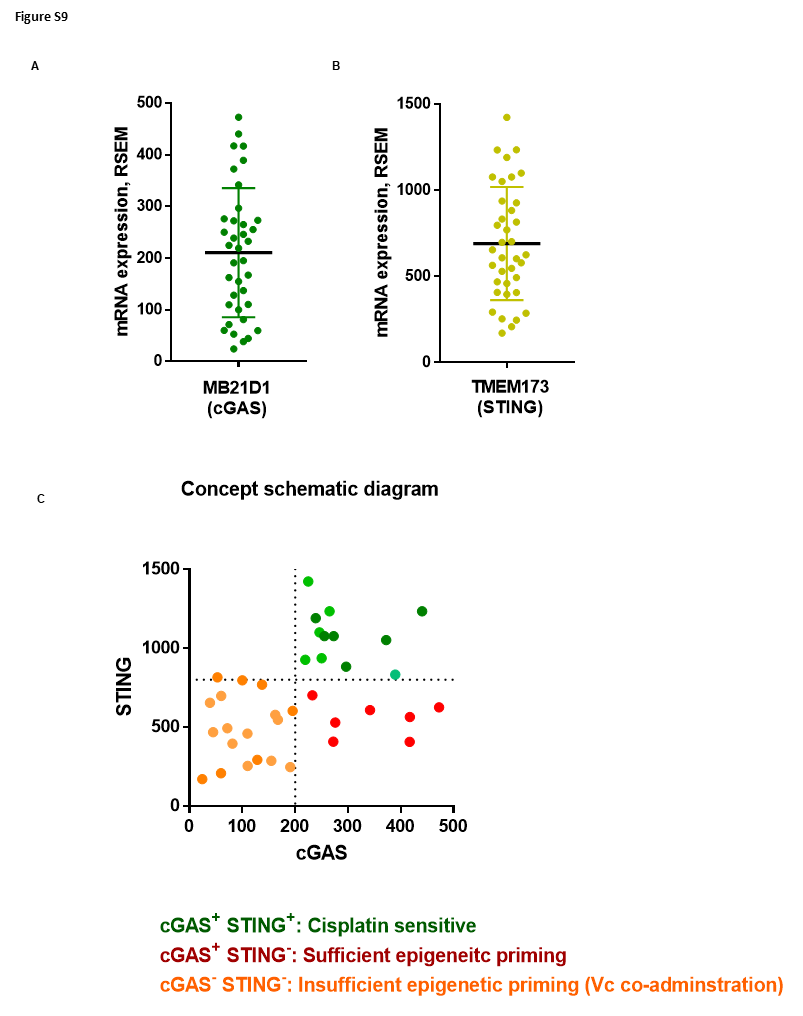


# Figure S9

# (A-B) MB21D1 and TMEM173, encoded cGAS and STING respectively, normalized expression level in 37 DLBCL patients (TCGA, PanCancer Atlas), (C) Concept schematic diagram to stratify DLBCL patients according to cGAS/STING expression level. The dots represent TCGA DLBCL patients’ cGAS and STING expression levels.

#
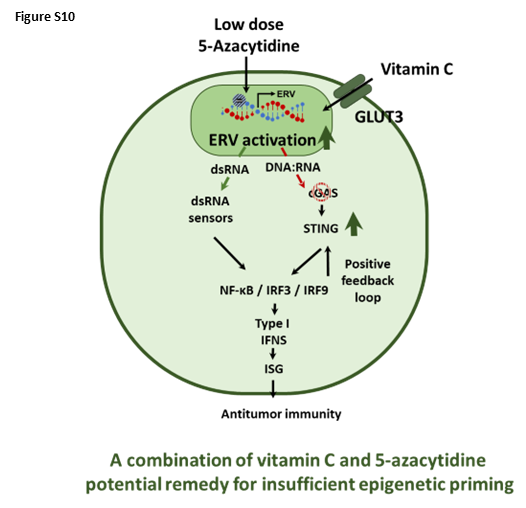


# Figure S10

# Vitamin C showed the potential to improve insufficient epigenetic priming, and this effect was dependent on the GLUT3 level.

# Table S1. Antibodies

| **Antibody** | **Company** | **Catalog number** |  |  |
| --- | --- | --- | --- | --- |
| p-H2AX | Cell Signaling | #9718 |  |  |
| GAPDH | Cell Signaling | #5174 |  |  |
| 5mC | Abcam | ab10805 |  |  |
| 5hmc | Abcam | ab214728 |  |  |
| MDA-5 | Cell Signaling | #5321 |  |  |
| Rig-1 | Cell Signaling | #3743 |  |  |
| TLR-3 | Cell Signaling | #6961 |  |  |
| cGAS | Cell Signaling | #15102 |  |  |
| STING | Cell Signaling | #13647 |  |  |
| TLR9 | Cell Signaling | #2254 |  |  |
| P-IRF3 | Cell Signaling | #29047 |  |  |
| IRF3 | Cell Signaling | #11904 |  |  |
| P-IκBα | Cell Signaling | #2859 |  |  |
| IκBα | Cell Signaling | #4814 |  |  |
| ADAR1L | Cell Signaling | #32136 |  |  |
| Anti-rabbit IgG, HRP-linked Antibody | Cell Signaling | #7074 |  |  |
| Anti-mouse IgG, HRP-linked Antibody | Cell Signaling | #7076 |  |  |

# Table S2. RT–qPCR primer sequences

| **Gene** | **Forward (5'-3')** | **Reverse (5'-3')** |
| --- | --- | --- |
| GAPDH | CTC CTC CAC CTT TGA CGC TG | TCC TCT TGT GCT CTT GCT GG |
| MLT1B | TGC CTG TCT CCA AAC ACA GT | TAC GGG CTG AGC TTG AGT TG |
| ERVL | ATA TCC TGC CTG GAT GGG GT | GAG CTT CTT AGT CCT CCT GTG T |
| MER4D | CCC TAA AGA GGC AGG ACA CC | TCA AGC AAT CGT CAA CCA GA |
| ERV 9-1 | TCT TGG AGT CCT CAC TCA AAC TC | ACT GCT GCA ACT ACC CTT AAA CA |

#

# Table S3. siRNA sequences

| **siRNA name** | **Duplex sequence (5'-3')** |
| --- | --- |
| cGAS siRNA#1 (MB21D1 #1) Sense | GAU UUC UGC ACC UAA UGA AUU |
| cGAS siRNA#1 (MB21D1 #1) Antisense | UUC AUU AGG UGC AGA AAU CUU |
| cGAS siRNA#2 (MB21D1 #2) Sense | CUA AGA UGC UGU CAA AGU UUU |
| cGAS siRNA#2 (MB21D1 #2) Antisense | AAC UUU GAC AGC AUC UUA GUU |
| STING siRNA#1 (TMEM173 #1) Sense | CAU AUU ACA UCG GAU AUC UUU |
| STING siRNA#1 (TMEM173 #1) Antisense | AGA UAU CCG AUG UAA UAU GUU |
| STING siRNA#2 (TMEM173 #2) Sense | GGA UUC GAA CUU ACA AUC AUU |
| STING siRNA#2 (TMEM173 #2) Antisense | UGA UUG UAA GUU CGA AUC CUU |
| Negative control Sense | CCU CGU GCC GUU CCA UCA GGU AGU U |
| Negative control Antisense | CUA CCU GAU GGA ACG GCA CGA GGU U |
